# Aflatoxins and Fumonisins: Assessment Methods, Biomarkers of Exposure, Modified Forms, Co-Exposure, and Impact on Human Health

**DOI:** 10.3390/molecules31132279

**Published:** 2026-06-29

**Authors:** Leakey Kuloba, Andrzej Wasik

**Affiliations:** Department of Analytical Chemistry, Faculty of Chemistry, Gdańsk University of Technology, 11/12 G. Narutowicza Str., 80-233 Gdańsk, Poland; leakey.kuloba@pg.edu.pl

**Keywords:** HRMS, modified mycotoxins, aptamers, green chemistry, co-exposure, human biomonitoring

## Abstract

Aflatoxins and fumonisins are two of the most prevalent and toxicologically significant mycotoxins contaminating global food supplies, particularly maize and groundnuts. Although several regulated mycotoxins contribute to food safety concerns, this review focuses on aflatoxins and fumonisins because they frequently co-occur in maize and maize products. Their widespread prevalence, distinct toxicological mechanisms, and combined health effects necessitate an integrated exposure and risk assessment. This review critically evaluates the current state of exposure assessment and its implications for human health. We examine the evolution of sample preparation techniques, highlighting the transition from traditional liquid–liquid extraction to advanced approaches such as QuEChERS and green extraction technologies that can handle the divergent physicochemical properties of lipophilic aflatoxins and hydrophilic fumonisins. Analytical methods are compared, from the robust but limited HPLC-FLD to the multi-analyte capabilities of LC-MS/MS and the emerging potential of aptamer-based biosensors. Furthermore, the review addresses the critical challenge of modified mycotoxins that evade routine detection yet may contribute to total toxicity. By synthesizing data on biomarkers of exposure and the mechanisms of co-exposure, we discuss the complex interplay between these toxins in the etiology of hepatocellular carcinoma and neural tube defects. The review concludes that mitigating the public health burden of mycotoxins requires a holistic strategy that integrates HRMS for non-targeted analysis with human biomonitoring to capture the accurate individual-level exposure.

## 1. Introduction

Mycotoxins are fungal secondary metabolites that occur naturally and can contaminate food and animal feeds. The major mycotoxins impacting human health are aflatoxins, fumonisins, ochratoxin A, zearalenone, nivalenol, and deoxynivalenol [1]. A growing concern exists for aflatoxins and fumonisins due to their high toxicity, widespread prevalence, and the potential for co-contamination in food crops such as maize and peanuts [2]. Because human exposure to these toxins typically occurs simultaneously rather than in isolation, assessing their combined toxicological effects remains a critical challenge for comprehensive risk characterization.

Major food products affected by aflatoxins include maize, wheat, rice, sorghum, pistachio, almond, groundnuts, tree nuts, figs, cottonseed, and spices. Aflatoxin M_1_ and aflatoxin M_2_ are metabolites of aflatoxin B_1_ and B_2_, respectively. They are found in milk and dairy products [3]. Fumonisins contaminate corn, corn-derived products, rice, sorghum, and asparagus [4].

Exposure to aflatoxins and fumonisins is associated with severe health effects. Aflatoxins affect the liver, causing acute symptoms such as nausea, vomiting, and liver failure at high doses. Chronic exposure is linked to liver cancer, immune suppression, growth retardation, and reproductive issues [5]. Exposure to fumonisins is associated with a wide range of health risks, such as sphingolipid metabolism disruption, which can lead to neural tube defects in developing fetuses. They have also been linked to hepatotoxicity, nephrotoxicity, immunotoxicity, and gastrointestinal disturbances. Chronic exposure is suspected to contribute to esophageal cancer in high-risk populations and may increase sensitivity to other infections due to immune suppression [6].

In affected regions such as Sub-Saharan Africa and South East Asia, legislation on aflatoxins and fumonisins is either less stringent or poorly enforced compared with the European Union. The gaps in harmonized standards, monitoring infrastructure, and farmers’ training lead to higher contamination levels and trade barriers [7]. The EU enforces strict maximum limits and uses the Rapid Alert System for Food and Feed (RASFF) for compliance. However, differences between EU regulatory limits and Codex Alimentarius standards, which are adopted by several countries, may complicate international trade and regulatory harmonization. In several commodity categories, the EU applies more stringent maximum levels than Codex Alimentarius, reflecting differences in risk assessment approaches and consumer protection objectives [8,9]. A summary of the maximum limits for selected products is presented in Table 1. The data is compiled from Commission Regulation (EU) 2023/915 [9] and Codex General Standard for Contaminants and Toxins in Food and Feed (CXS 193-1995) [8].

This review provides a comprehensive overview of aflatoxins and fumonisins, emphasizing their co-occurrence, exposure assessment, and human health implications. It examines their chemical and structural characteristics, detection and quantification methods, exposure biomarkers, and applications in human biomonitoring. Special attention has been paid to emerging challenges, including modified mycotoxins, co-exposure, and potential toxicological interactions. Finally, the review highlights recent analytical advances and outlines future research recommendations for improving risk characterization and regulatory monitoring.

## 2. Chemical and Structural Characteristics

Aflatoxins and fumonisins are secondary metabolites produced by the *Aspergillus* and *Fusarium* species, respectively [10]. Their chemical and structural properties influence their detection, identification, and mode of action. These characteristics are vital for developing analytical methods and designing mitigation strategies.

### 2.1. Aflatoxins

Aflatoxins are produced by *Aspergillus flavus* and *Aspergillus parasiticus* [11]. A few studies, including Habibi and Afzali [12], have reported that *Aspergillus nomius* and *Aspergillus tamarii* also produce aflatoxins. Over 18 types of aflatoxins have been investigated according to studies by Abrehame et al. and Yang et al. [13,14]. However, research has primarily focused on aflatoxin B_1_, B_2_, G_1_, G_2_, and aflatoxin M_1_—a metabolite of aflatoxin B_1_—because of their health implications [14,15].

**Figure 1:**
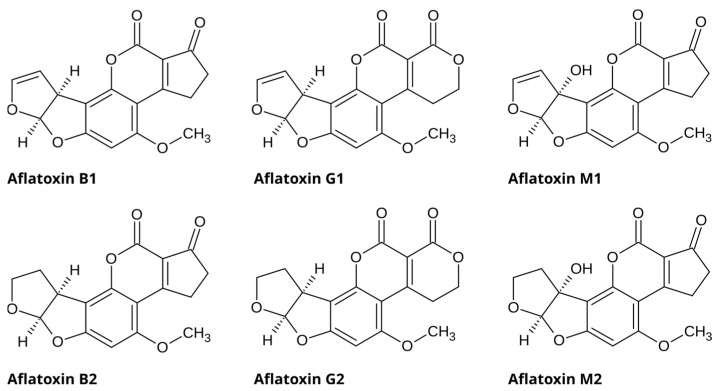
Chemical structures of selected aflatoxins.

The chemical structure of an aflatoxin consists of a bifuran moiety attached to a coumarin nucleus and either a pentanone or lactone ring. Aflatoxin B_1_ and B_2_ contain the pentanone ring, and fluoresce blue under UV light, whereas aflatoxin G_1_ and G_2_ have a lactone ring and fluoresce green under UV light, hence the letters “B” and “G” in their names, respectively. Aflatoxin M_1_ found in milk and dairy products has a pentanone ring, with the letter “M” indicating its origin from milk [3,14]. The C8=C9 double bond present in aflatoxin B_1_, G_1_, and M_1_ is absent in aflatoxin B_2_, G_2_, and M_2_ [14,16,17]. Figure 1 shows the chemical structures of the most prevalent aflatoxins.

Aflatoxins are colorless to pale yellow crystals with low molecular weight. They are thermally stable and can withstand high temperatures during storage, processing, and cooking. They are soluble in organic solvents such as acetonitrile, benzene, chloroform, and methanol, but are insoluble in non-polar solvents, and moderately soluble in water [18]. Aflatoxins remain stable within the pH range of 3 to 10, but become unstable in the presence of oxygen [14].

### 2.2. Fumonisins

**Figure 2:**
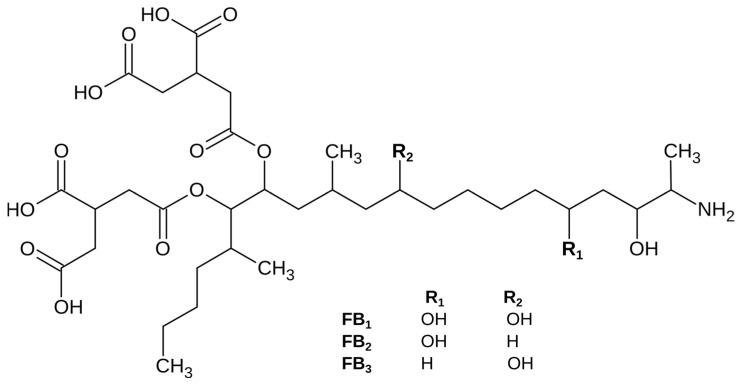
Chemical structures of fumonisins B.

More than 18 *Fusarium* species produce fumonisins, with *Fusarium verticillioides*, *Fusarium proliferatum*, and *Fusarium subglutinans* being the three major producers [4,19]. Over 20 types of fumonisins have been identified and grouped into the A, B, C, D, P, and L series based on the length of the carbon backbone and nitrogen functional group [4,20]. The B series is the most prevalent, with FB_1_ typically dominating in contaminated commodities. While research predominantly focuses on FB_1_, the simultaneous presence of FB_2_ and FB_3_ significantly contributes to overall human dietary exposure [6]. The chemical structures of the B series are shown in Figure 2.

Fumonisin structures consist of different polyhydric alcohols and tricarboxylic acids. Their most distinctive property is the lack of cyclic structure and solubility in water [13]. Chemically, fumonisins resemble sphingosine and sphinganine found in sphingolipids [20,21]. Fumonisins are thermally stable and can survive at temperatures up to 150 °C [22].

### 2.3. Chemical Transformations

Both aflatoxins and fumonisins undergo several chemical transformations during cooking and storage, as summarized in Table 2.

## 3. Analytical Methods for Detection and Quantification

Aflatoxins and fumonisins differ significantly in terms of their physicochemical properties—aflatoxins are moderately lipophilic and fluorescent, while fumonisins are highly polar and non-fluorescent. These differences affect sample preparation, sensitivity, and selectivity of analytical methods. This section reviews the analytical methods used to detect and quantify aflatoxins and fumonisins, highlighting recent trends in analytics, limitations, and methodological considerations.

### 3.1. Sample Pretreatment

Sample preparation and extraction are fundamental steps in analytical method development for the detection and quantification of mycotoxins. They help to create representative samples, reduce matrix effects, and concentrate analytes to detectable levels. However, the extraction of aflatoxins and fumonisins presents distinct analytical challenges due to their differing physicochemical properties. While aflatoxins are often extracted using organic solvents, fumonisins, being more polar, often require acidified extraction methods and are more susceptible to matrix effects. In addition, matrix-associated fumonisins may not be fully recovered by conventional extraction methods, leading to underestimation of exposure [32]. Therefore, the selection of extraction and clean-up methodologies must balance recovery, selectivity, robustness, and suitability for routine regulatory monitoring. Characteristics of a suitable sample preparation procedure include low cost, minimal sample and solvent consumption, effective reduction in matrix interferences, high reproducibility, and acceptable analyte recovery. Table 3 provides a comprehensive analysis of extraction and clean-up methodologies used for aflatoxin and fumonisin analysis. The reported performance metrics originate from different studies, matrices, analytes, and validation protocols. Therefore, direct comparisons between methods should be interpreted with caution.

#### 3.1.1. Liquid–Liquid Extraction/Partitioning (LLE)

Liquid–liquid extraction remains one of the most widely used extraction techniques due to its simplicity and applicability to a broad range of food matrices. However, extraction efficiency is highly dependent on the solvent composition and the analyte’s physicochemical properties. Compared with aflatoxins, fumonisins present greater extraction challenges because their high polarity requires acidified solvents and may increase susceptibility to matrix effects [35]. Furthermore, conventional LLE procedures are labor-intensive, consume relatively large volumes of organic solvents, and may co-extract matrix components [33,34]. These limitations have led to the development of miniaturized alternatives, such as dispersive liquid–liquid microextraction (DLLME), which reduce solvent consumption and improve extraction efficiency while maintaining acceptable analytical performance [34,36,37].

#### 3.1.2. Solid–Liquid Extraction (SLE)

Solid–liquid extraction is commonly used to extract aflatoxins and fumonisins due to its simplicity and low cost. Polar organic solvents or mixtures such as acetonitrile/water, methanol/water, and acetonitrile/methanol/water are typically employed in various ratios, with solvent selection dependent on both the matrix and target myotoxin [32].

Aflatoxins are moderately lipophilic and soluble in polar organic solvents. In contrast, fumonisins are highly polar and thus extract poorly with organic-only solvents. The inclusion of water and pH adjustment to protonate the amino moiety often improves the extraction efficiency. Despite its widespread use, SLE has several limitations, including high solvent consumption, labor intensity, and the co-extraction of matrix components that may interfere with subsequent analysis [38]. Additionally, the extraction efficiency of fumonisins can be affected by matrix-associated forms, leading to the underestimation of exposure. Therefore, standard SLE must be combined with chemical or enzymatic hydrolysis to liberate these bound fractions prior to chromatographic analysis.

#### 3.1.3. Solid-Phase Extraction (SPE)

Solid-phase extraction has proven to be a better alternative to the LLE because it reduces solvent consumption, shortens extraction time, and improves sample clean-up efficiency [44]. The method is widely used owing to its high selectivity, ability to reduce matrix interferences, and compatibility with chromatographic systems. SPE involves loading the sample onto a cartridge containing the sorbent material, where target analytes are retained while the unwanted matrix components are washed away. The retained analytes are eluted from the cartridge and analyzed by an appropriate analytical method. Several types of sorbents have been used in SPE, including C18- and C8-modified silica, hydrophilic–lipophilic balance (HLB) polymers, mixed-mode/anion-exchange (MAX), mixed-mode/cation-exchange (MCX), and weak anion exchange (WAX) resins [67]. SPE is remarkably effective for relatively nonpolar mycotoxins such as aflatoxins; however, fumonisins may require specialized sorbents, such as ion-exchange and mixed-mode materials, to improve retention and recovery due to their high polarity and susceptibility to matrix effects [40]. The conventional SPE’s limitations, such as poor selectivity in complex food matrices, low capacity, and limited reusability, have led to the emergence of miniaturized SPE techniques such as dispersive SPE (d-SPE), micro-SPE (µ-SPE), solid-phase microextraction (SPME), magnetic SPE (MSPE), stir-bar sorptive extraction (SBSE), and pipette-tip SPE (PT-SPE) [67,68].

Dispersive SPE improves analyte–sorbent interaction by dispersing the sorbent directly into the extract, resulting in faster extraction and improved clean-up efficiency [39]. The technique has been successfully applied to the determination of aflatoxins, fumonisins, zearalenone, and deoxynivalenol in food and feed matrices, generally achieving satisfactory recoveries, reduced matrix interference, and compatibility with LC-MS/MS-based methods [40,69,70]. The performance of d-SPE strongly depends on sorbent selection, particularly in multi-mycotoxin applications where analytes exhibit diverse physicochemical properties.

Micro-SPE uses a small amount of sorbent enclosed in a porous membrane, enabling extraction, clean-up, and preconcentration in a single step. The membrane protects the sorbent from loss due to interactions with extraneous matter, making the method suitable for complex samples [41]. Reported applications have demonstrated satisfactory recoveries and sensitivity for aflatoxin determination when coupled with UHPLC-MS/MS systems [42]. However, analytical performance remains highly dependent on sorbent characteristics and membrane stability.

Solid-phase microextraction was initially developed for the analysis of volatile compounds, but has subsequently been adapted for liquid chromatographic analysis of semi-volatile and non-volatile compounds, including aflatoxins [34]. The technique uses a fiber coated that selectively extracts analytes from the sample matrix. Various functionalized coatings have demonstrated satisfactory recoveries, sensitivity, and precision for aflatoxin analysis [43]. However, fumonisins are highly polar, water-soluble compounds that partition poorly in conventional SPME coatings. Their extraction often requires derivatization or specialized coatings such as ionic liquids, molecularly imprinted polymers, or metal–organic frameworks. Consequently, SPME has been applied more extensively for the extraction of aflatoxins than fumonisins [44].

Magnetic SPE has emerged as a rapid, high-throughput alternative to conventional SPE by using magnetic sorbents that can be easily isolated with an external magnetic field [45]. Functionalized magnetic nanoparticles improve extraction selectivity and reduce extraction time. Applications have demonstrated good recoveries for multiple mycotoxins, including aflatoxins and fumonisins, while enabling shorter pretreatment times and reduced labor intensity compared with conventional cartridge-based SPE [46,71]. Nevertheless, matrix effects and sorbent-specific performance remain important considerations during method optimization.

Stir-Bar Sorptive Extraction uses a sorbent-coated magnetic stir bar that simultaneously agitates the sample and extracts analytes [47]. Conventional SBSE relies on hydrophobic polydimethylsiloxane (PDMS) coatings, which limit its applicability to polar analytes such as fumonisins and moderately polar analytes such as aflatoxins. Thus, molecularly imprinted polymers and other advanced coatings have been investigated to overcome these limitations [48,49]. Despite its low solvent consumption and operational simplicity, SBSE remains less widely used than conventional SPE methods.

Pipette-tip solid-phase extraction miniaturizes conventional SPE by packing sorbent material directly into a micropipette tip [49]. Compared with conventional SPE, this technique offers superior operational efficiency through reduced solvent consumption, reduced sample volume, shorter extraction time, and the feasibility of on-site execution, making it suitable for rapid screening applications. However, as a non-exhaustive extraction method, its analytical performance depends on the sorbent’s selectivity and physicochemical characteristics [72]. Applications using graphene-based sorbents have demonstrated satisfactory recoveries and sensitivity for aflatoxin analysis, although routine implementation remains limited [50].

Collectively, miniaturized SPE methods reduce solvent consumption, shorten extraction times, and improve analytical throughput relative to conventional SPE. However, many remain at the trial stage and have not achieved the degree of validation, standardization, and routine laboratory implementation that conventional SPE methods have. Their performance can vary considerably depending on the matrix composition, sorbent characteristics, and analyte polarity, particularly for highly polar fumonisins. Therefore, method selection should be guided not only by analytical performance but also by validation status, matrix suitability, and intended regulatory application.

#### 3.1.4. Energy-Assisted Extraction

Energy-assisted extraction techniques utilize external energy sources to enhance mass transfer and disrupt sample matrices, thereby improving extraction efficiency, reducing solvent consumption, and shortening extraction time compared with conventional extraction methods [34]. These techniques include ultrasound-assisted extraction (UAE), microwave-assisted extraction (MAE), pressurized liquid extraction (PLE), and supercritical fluid extraction (SFE).

Ultrasound-assisted extraction relies on acoustic cavitation, whereby the formation and collapse of microbubbles generate shear forces that disrupt sample matrices and facilitate analyte release [73]. UAE has been successfully applied to fumonisin analysis in maize, providing rapid extraction, satisfactory validation parameters, and compatibility with LC-MS/MS workflows [51].

Microwave-assisted extraction uses microwave energy to increase analyte solubility and diffusion, resulting in faster extraction and lower solvent consumption [52]. The technique has been successfully applied to the simultaneous extraction of mycotoxins, including aflatoxins and fumonisins, with reported recoveries and detection limits suitable for trace-level analysis [53].

Pressurized liquid extraction, also referred to as accelerated solvent extraction (ASE), uses elevated temperatures and pressure to enhance analyte solubility and mass transfer in solid and semi-solid matrices. Compared with MAE, PLE offers distinct advantages of automation and in-cell filtration, thereby eliminating the post-extraction clean-up step, although these benefits are offset by the higher cost of instrumentation and more labor-intensive cell preparation [54]. PLE has been successfully combined with green solvents such as deep eutectic solvents for aflatoxin determination in rice and other cereal matrices, with satisfactory recoveries and detection limits suitable for trace-level analysis [55].

Supercritical fluid extraction utilizes solvents at supercritical temperatures and pressure to achieve gas-like diffusivity and liquid-like viscosity, thereby enhancing mass transfer and matrix penetration. Using supercritical CO_2_, this technique selectively extracts nonpolar analytes and eliminates the need for organic solvents, providing an environmentally friendly and cost-effective method for mycotoxin analysis [56]. However, since supercritical CO_2_ lacks the polarity to extract water-soluble toxins on its own, polar modifiers such as water or methanol are added [57]. Consequently, while SFE is generally more suitable for relatively non-polar analytes such as aflatoxins, fumonisins typically require polar modifiers to ensure complete recovery.

Although energy-assisted extraction techniques offer faster extraction, reduced solvent consumption, and improved analytical throughput, their performance strongly relies on matrix composition and analyte properties. In particular, the high polarity of fumonisins may require method-specific optimization to achieve satisfactory recoveries. In addition, the intensive matrix disruption associated with these techniques can increase co-extraction of interfering compounds, often requiring additional clean-up procedures. Therefore, despite their promising analytical performance, high equipment cost and limited standardization currently restrict widespread adoption in routine analysis [34].

#### 3.1.5. Immuno-Affinity Column Clean-Up

Immuno-affinity column (IAC) clean-up relies on highly specific antigen–antibody interactions and remains one of the most widely used sample clean-up methods for mycotoxin analysis. Owing to their high selectivity, IACs effectively remove matrix interferences and are incorporated into numerous official and standardized methods for the determination of aflatoxins and other regulated mycotoxins [34,58].

IAC-based methods have demonstrated excellent analytical performance for aflatoxin determination in cereals. For example, methanol-water extraction followed by IAC clean-up and HPLC-MS/MS analysis achieved recoveries of 86–92%, and detection limits well below regulatory thresholds for aflatoxins in rice [59]. IAC methods have also been applied to fumonisin analysis. Anumudu [74] reported a reusable immunoaffinity clean-up method coupled with UHPLC/ESI-MS/MS for the determination of fumonisin B_1_, B_2_, and B_3_ in maize. Although the method achieved high sensitivity, substantial matrix suppression reduced recoveries in real samples, highlighting the greater analytical challenges associated with fumonisin determination compared with aflatoxins.

Compared with emerging clean-up technologies, IAC remains the standard method for selective mycotoxin clean-up in many routine and regulatory analyses. Its ability to reduce matrix effects and improve method sensitivity has supported its incorporation into numerous official analytical methods. However, the method is less suited to broad-spectrum multi-mycotoxin analysis because antibody specificity limits the range of analytes that can be analyzed simultaneously. As a result, IAC is increasingly complemented by LC-MS/MS-based multi-analyte workflows and alternative clean-up methods for comprehensive exposure assessment.

#### 3.1.6. QuEChERS

The Quick, Easy, Cheap, Effective, Rugged, and Safe (QuEChERS) method was originally developed for the analysis of multi-pesticide residues in fruit and vegetable samples. Over time, it has become a popular sample preparation technique for other matrices and analytes, such as mycotoxins, because of its versatility and throughput [60,61,62]. The QuEChERS approach involves an extraction step followed by a clean-up step. An initial solid–liquid extraction is performed using acetonitrile, followed by salting out using MgSO_4_ and NaCl to facilitate water removal and phase separation. The supernatant then undergoes a dispersive SPE clean-up step in which PSA sorbent and MgSO_4_ are added. PSA selectively removes acidic matrix interferences such as lipids and sugars, while MgSO_4_ eliminates the remaining water content. This yields a purified extract suitable for immediate chromatographic analysis [60,62]. Unlike traditional extraction techniques that target specific analyte classes, the QuEChERS method can simultaneously analyze aflatoxins and fumonisins because its single-step procedure accommodates both the lipophilic nature of aflatoxins and the hydrophilic, ionic nature of fumonisins. Consequently, it reduces time and solvent consumption compared with running parallel extraction steps [63].

The simultaneous extraction of aflatoxins and fumonisins faces a chemical challenge that cannot be addressed by the standard QuEChERS method. The tricarballylic acid side chains in fumonisins B_1_ and B_2_ make them anionic and highly soluble in water, resulting in poor recovery from the organic phase. Therefore, the QuEChERS method is modified by introducing an acidification step: 0.3–3% formic acid is added to the acetonitrile (extraction solvent) to suppress ionization of fumonisins. This ensures that they partition effectively into the organic layer along with the non-polar aflatoxins [75]. The d SPE clean-up step is critical for fumonisin recovery. The standard QuEChERS method employs PSA and C18 to remove matrix interferences such as sugars and lipids. However, PSA acts as a weak anion exchanger that binds to the acidic carboxyl groups of fumonisins, leading to loss of recovery [32]. An additional challenge in fumonisin analysis is the occurrence of matrix-associated forms that remain bound to food macromolecules and are not completely recovered by conventional extraction methods. Since QuEChERS primarily targets extractable fumonisins, these bound forms may escape detection unless hydrolysis or other release mechanisms are incorporated. As a result, hidden fumonisins pose a challenge in exposure assessment and may contribute to the underestimation of total dietary intake.

Several modified QuEChERS approaches have been developed to overcome limitations associated with conventional PSA-based clean-up. Enhanced matrix removal lipid (EMR lipid) sorbents have demonstrated improved lipid removal and reduced matrix effects in high-fat commodities such as nuts and animal feeds, while maintaining satisfactory recoveries and high sensitivities [64,76]. Similarly, metal–organic frameworks (MOFs), magnetic nanomaterials, graphene-based sorbents, and multi-walled carbon nanotubes (MWCNTs) have been investigated as alternative clean-up materials because of their selective adsorption properties [63,65,66,77,78,79]. These materials provide improved matrix clean-up, low detection limits, and satisfactory recoveries. However, most remain at the experimental stage and require further validation before widespread adoption in routine regulatory laboratories.

Recent adaptations of the QuEChERS methodology, including p-QuEChERS (FATChERS) and QuEChERSERS, have been developed to improve analyte extraction from complex matrices [75]. These approaches employ modified solvent systems, cryogenic processing, or additional clean-up steps to improve analyte recovery and reduce variability. Although their application to mycotoxin analysis remains limited, they may offer future opportunities to improve the extraction of challenging mycotoxins in complex food matrices.

Owing to its simplicity, low solvent consumption, and compatibility with multi-mycotoxin LC-MS/MS workflows, QuEChERS has become one of the most widely used extraction methods for the simultaneous determination of aflatoxins and fumonisins. Nevertheless, method performance remains highly dependent on matrix composition, clean-up sorbent selection, and optimization of fumonisin recovery. Therefore, matrix-specific validation remains essential, particularly when highly polar fumonisins or hidden forms are targeted.

### 3.2. Detection and Quantitative Techniques

The accurate determination of aflatoxins and fumonisins in food matrices remains analytically challenging due to their different physicochemical properties, complexity of food matrices, and the occurrence of modified or matrix-associated forms. While aflatoxins are relatively hydrophobic and naturally fluorescent, fumonisins are highly polar compounds that often require derivatization or advanced mass spectrometric detection. Consequently, analytical methods must be carefully selected and validated based on target analytes, matrix characteristics, sensitivity, and intended application. Broadly, these techniques are categorized into confirmatory chromatographic methods and rapid immunochemical screening approaches [58]. Table 4 provides an overview of chromatographic and immunochemical techniques used for aflatoxin and fumonisin analysis. However, the reported performance characteristics originate from different studies, analytes, matrices, and validation protocols; therefore, direct comparison between methods should be interpreted with caution.

#### 3.2.1. Chromatographic Techniques

Chromatographic techniques remain the reference methods for determining aflatoxins and fumonisins due to their high sensitivity, selectivity, and ability to meet stringent regulatory limits. Their evolution, from simple planar methods to advanced hyphenated chromatographic methods, reflects the growing need for reliable quantification of chemically diverse mycotoxins in complex food matrices. Although chromatographic methods generally require more extensive sample preparation, specialized instrumentation, and skilled operators than rapid screening assays, they provide the analytical performance necessary for confirmatory analysis and regulatory monitoring [80].

Thin-Layer Chromatography (TLC) was among the earliest validated methods for aflatoxin analysis and remains widely used in cost-constrained settings due to its cost-effectiveness [34]. Aflatoxins can be detected directly due to their natural fluorescence, whereas fumonisins require derivatization prior to analysis due to the absence of native fluorescent or UV-active functional groups [81,82]. Despite advancements in TLC, such as high-performance TLC (HPTLC), two-dimensional TLC (2D TLC), and over-pressured thin-layer chromatography (OPTLC), the technique suffers from relatively low sensitivity, limited automation, and reduced reproducibility compared with modern liquid chromatographic methods. Thus, TLC is mainly used for preliminary screening rather than confirmatory analysis [34].

High Performance Liquid Chromatography (HPLC) is widely used for mycotoxin analysis because of its flexibility, robustness, and compatibility with various detection systems [83]. Reversed-phase HPLC coupled with ultraviolet–visible (UV-Vis) or fluorescence detection (FLD) has been extensively used for aflatoxin determination. However, the simultaneous analysis of aflatoxins and fumonisins using these detectors remains analytically challenging due to their different physicochemical properties [83,84,85,86]. Aflatoxins contain naturally fluorescent and UV-active chromophores that enable detection by UV-Vis and FLD detectors. In contrast, fumonisins lack both UV chromophore and fluorescence, making direct detection difficult [83,85,86,87]. Therefore, fumonisin analysis by HPLC-UV-Vis or HPLC-FLD typically requires derivatization with reagents such as o-phthalaldehyde (OPA), whereas aflatoxins may require pre- or post-column derivatization to achieve adequate sensitivity [83,86,87]. These additional steps increase analytical complexity, extend sample processing time, and may introduce variability. Additionally, fumonisins are highly polar and susceptible to matrix effects; therefore, they often require matrix-specific extraction and clean-up procedures to achieve acceptable recoveries.

**Table 4:** Comprehensive analysis of analytical techniques for aflatoxin and fumonisin determination.

Technique	Selectivity	Sensitivity	Suitability for Simultaneous Determination of Aflatoxins and Fumonisins	Sample Preparation Complexity	Throughput	Advantages/Limitations	References
TLC	Low to moderate	LOD: 0.81–1.0 µg·kg^−1^ (AFs)	Limited; simultaneous separation is difficult	Low	Moderate	Simple, cheap, but poor resolution and sensitivity	[81,88]
HPLC-UV-Vis	Moderate to good	LOD: 0.3–1.0 µg·kg^−1^ (AFs); fumonisins typically require derivatization for comparable sensitivity	Possible but limited by UV overlap of analytes	Moderate	High	Requires careful method development	[83,89]
HPLC-FLD	High	LOD: 0.10–0.11 µg·kg^−1^ (AFs); generally less sensitive for fumonisins	Less suitable for fumonisins	Moderate	High	Fumonisins need derivatization	[83,87,90]
LC-MS/MS	Very high	LOD: 0.013–3.33 ng·g^−1^ (AFs and FBs)	Excellent for simultaneous detection of aflatoxins, fumonisins, and other co-occurring mycotoxins	High	High	High initial cost of equipment, highly trained operator required	[91,92,93]
ELISA	High for single toxin	LOD: 5.5–6.6 ng·kg^−1^ (AFB_1_), 20 µg·kg^−1^ (FBs)	Limited for simultaneous detection of multi-mycotoxins	Low to moderate	Very high	Rapid screening tool, susceptibility to antibody cross-reactivity, and limited multiplexing capability	[58,94,95,96,97]
LFA	Moderate	LOD: 0.71–1.4 µg·kg^−1^ (AFs), 4000 µg·kg^−1^ (FBs)	Limited for simultaneous detection of multi-mycotoxins	Low	Very high	Rapid, portable and cost effective, primarily qualitative or semi-quantitative	[98,99,100,101]

TLC: Thin-layer chromatography; HPLC-UV-Vis: High-performance liquid chromatography with ultraviolet–visible detection; HPLC-FLD: High-performance liquid chromatography with fluorescence detection; LC-MS/MS: Liquid chromatography–tandem mass spectrometry; ELISA: Enzyme-linked immunosorbent assay; LFA: Lateral flow assay. Note: The reported sensitivity values were obtained from different studies, analytes, matrices, and validation protocols; therefore, direct comparison between methods should be interpreted with caution.

Due to these challenges, liquid chromatography coupled with tandem mass spectrometry (LC-MS/MS) has become the preferred method for the simultaneous determination of aflatoxins and fumonisins. The method combines efficient chromatographic separation with highly selective mass spectrometric detection, enabling simultaneous quantification of structurally diverse mycotoxins without the need for complex derivatization [38,58,85,102]. The use of multiple reaction monitoring (MRM) provides high sensitivity and selectivity, allowing quantification at concentrations that meet or exceed regulatory requirements [102,103].

One major advantage of LC-MS/MS is its suitability for multi-mycotoxin analysis. Co-contamination of aflatoxins and fumonisins is frequently reported, particularly in maize and maize products. Therefore, analytical methods capable of detecting both mycotoxins within a single run are necessary for exposure assessment and regulatory monitoring [92,93]. In addition, LC-MS/MS facilitates the simultaneous determination of other regulated and emerging mycotoxins, thereby improving laboratory efficiency and reducing costs [104,105].

Despite its excellent analytical performance, LC-MS/MS remains susceptible to matrix effects caused by co-eluting compounds that may suppress or enhance ionization efficiency. To compensate for these effects, several calibration strategies have been developed, such as matrix-matched calibration, standard addition, and the use of stable isotope-labeled internal standards [106,107]. While the stable isotope dilution assays generally provide the highest accuracy, their use is often limited by the high cost and availability of labeled standards.

Recent advances in extraction and clean-up methodologies have further strengthened the use of LC-MS/MS in multi-mycotoxin analysis. Techniques such as QuEChERS and IAC have improved sample purification and reduced matrix interferences, enabling reliable determination of aflatoxins and fumonisins across a wide range of food and feed matrices [108,109,110]. Despite the initial high cost of equipment and the need for specialized expertise, LC-MS/MS is still regarded as a reference method for the simultaneous determination of aflatoxins and fumonisins due to its high sensitivity, selectivity, and versatility [111].

#### 3.2.2. Immunochemical Methods

For routine monitoring of food and feed products, immunochemical methods offer rapid, sensitive, and relatively simple alternatives to chromatographic methods. These methods exploit the specific interactions between antibodies and their target mycotoxins, enabling selective recognition even in complex food matrices [58,112]. Compared with chromatographic methods, immunoassays generally require less sample preparation, lower capital investment, and shorter analysis times, making them suitable for large-scale screening applications. However, they are typically considered screening tools and often require confirmatory analysis by chromatographic methods when regulatory decisions are involved [58].

Enzyme-linked immunosorbent assay (ELISA) is the most widely used immunochemical technique for screening mycotoxins. ELISA kits available for aflatoxins and fumonisins achieve detection limits suitable for routine food safety monitoring [94,95,96,97]. Their analytical performance depends on antibody specificity, affinity, assay format, and matrix composition. Both monoclonal and polyclonal antibodies are used for the analysis of aflatoxins and fumonisins, with monoclonal antibodies typically offering improved selectivity and reproducibility [95,113].

While ELISA methods are widespread, they still present several analytical limitations. Matrix components may interfere with antibody binding, leading to signal suppression or enhancement, while antibody cross-reactivity with structurally related metabolites can compromise selectivity [96]. These challenges are more pronounced in fumonisins, where hydrolyzed, modified, or matrix-associated forms may interact with the antibodies differently. Therefore, ELISA results should be interpreted carefully, especially when assessing total exposure or compliance with regulatory limits. Chromatographic analysis is often required when positive samples are detected [58].

Lateral Flow Assays (LFAs), also called strip tests, complement ELISA by providing on-site qualitative or semi-quantitative assessment of mycotoxins. These portable devices employ antibody-based recognition on a fibrous nitrocellulose membrane and generate qualitative or semi-quantitative results within minutes [98,99]. Their ease of use, low cost, and minimum sample preparation requirements have promoted widespread adoption in field testing, grain storage facilities, and resource-limited settings.

Recent advances in immunochemical assay design, signal amplification strategies, and multiplex detection have improved the sensitivity and applicability of LFAs for mycotoxin screening [98,99]. These improvements have enhanced sensitivity and enabled simultaneous detection of multiple mycotoxins, including aflatoxins and fumonisins. However, LFAs generally exhibit lower sensitivity than chromatographic techniques and remain more susceptible to matrix effects and cross-reactivity. As a result, they are mainly used as rapid screening tools rather than confirmatory methods.

### 3.3. Recent Advances in Aflatoxin and Fumonisins Assessment

The complexity of mycotoxin contamination, including co-occurrence, low-level presence, and chemical modifications, drives continuous innovation in analytical techniques. Emerging technologies aim to enhance detection capabilities, address modified toxins, and reduce environmental impact [85,86].

#### 3.3.1. High Resolution Mass Spectrometry and Non-Targeted Analysis

High-resolution mass spectrometry (HRMS) instruments such as Orbitrap and Quadrupole time-of-flight (Q-TOF) deliver mass accuracies below 5 mg·kg^−1^ and resolving power up to 100,000 FWHM (Full width at half maximum), enabling unparalleled detection of mycotoxins and their metabolites. This precision facilitates non-targeted metabolomic approaches, where comprehensive profiling of food or biological samples can reveal known and emerging mycotoxins [114].

For aflatoxins and fumonisins, HRMS enables the detection of conjugated, modified, and previously uncharacterized metabolites that may escape routine targeted LC-MS/MS workflows. This capability is particularly important for matrix-associated fumonisins, which remain a major source of uncertainty in exposure assessment [115]. Retrospective data analysis is a key advantage, permitting reanalysis of archived datasets as new mycotoxin variants are characterized, thus enhancing surveillance and risk assessment [114,115,116]. In addition, HRMS enables targeted and non-targeted screening, allowing the identification of emerging mycotoxins without the need for analytical standards.

Recent studies employing HRMS have identified previously unreported aflatoxin and fumonisin metabolites, expanding the understanding of toxin biotransformation pathways [22,117,118]. Despite higher operational costs and the need for specialized expertise in operation and data interpretation, HRMS offers a significant upgrade over traditional targeted screening. These capabilities make HRMS particularly valuable for investigating modified and matrix-associated mycotoxins, whose occurrence and toxicological significance are increasingly recognized, but assessment remains challenging when using conventional targeted methods [119].

#### 3.3.2. The Challenge of Modified Mycotoxins

The accurate detection and quantification of aflatoxins and fumonisins are complicated by the occurrence of their modified forms. Modified mycotoxins originate primarily from plant defense mechanisms, in which enzymatic detoxification converts fungal toxins into more polar metabolites that are either stored in vacuoles or conjugated to cell-wall biopolymers. While plant-derived modifications are most prevalent, these compounds can also be generated by the fungi themselves, during human or animal metabolism, or through food processing. These transformations alter their physicochemical properties and can evade conventional detection [120,121]. These metabolites pose a significant analytical and toxicological challenge because they may hydrolyze in the gastrointestinal tract, releasing the parent toxins and contributing to underestimated exposure [121]. Modified forms of aflatoxins include hydroxylated metabolites such as AFM_1_, AFP_1_, and AFQ_1_, produced mainly in animals during biotransformation and excreted in urine or milk. While AFM_1_ is well recognized and regulated in dairy products, AFP_1_ and AFQ_1_ remain underexplored but contribute to overall exposure [122,123].

Modified fumonisins include hydrolyzed forms as well as masked and hidden forms that may be associated with carbohydrates, proteins, lipids, or other matrix components. Recently, attention has been paid to hidden or matrix-associated fumonisins, which pose an analytical challenge for assessing fumonisin exposure. Unlike free fumonisins, these forms are not readily extracted by conventional solvent-based methods and may therefore remain undetected during routine analysis, leading to underestimation of true contamination in food and feeds. Moreover, these matrix-associated forms can be released during food processing or gastrointestinal digestion, thereby contributing to toxin exposure, even if not detected in the original sample. Although toxicological data remain limited, increasing evidence suggests that these forms should be considered when evaluating dietary exposure and risk assessment [74,124].

Analytical detection of modified mycotoxins requires advanced, tailored methodologies capable of releasing or directly detecting bound forms. For fumonisins, alkaline hydrolysis and enzymatic digestion are commonly used to liberate matrix-associated toxins prior to chromatographic analysis [74,125,126]. In addition, LC-MS/MS and HRMS methods have become essential for the detection of free, modified, and previously uncharacterized fumonisin derivatives due to their high sensitivity and selectivity [127,128].

Regulatory authorities recognize the relevance of modified mycotoxins in exposure assessment. While aflatoxin M_1_ in milk is subject to regulatory limits, modified forms of fumonisins are not yet incorporated into regulatory frameworks due to limited occurrence, toxicological, and exposure data. Therefore, continued research is required to characterize these metabolites, clarify their health significance, and develop standardized methodologies that enable their inclusion in future risk assessment and monitoring programs [129].

#### 3.3.3. Aptamers for Mycotoxin Detection

Modern analytical methods for mycotoxin determination, such as HPLC and LC-MS/MS, exhibit high accuracy, sensitivity, and specificity; however, they remain hindered by elaborate sample preparation, high cost, and non-portable instrumentation. Recent developments, such as ELISA, facilitate rapid screening; however, they also present drawbacks, including antibody instability, matrix interference, cross-reactivity, and the need for confirmatory analysis. Consequently, this has led to the development of an aptamer-based method for detecting aflatoxins and fumonisins in food samples [130,131,132].

Aptamers are single-stranded nucleic acids (or peptides) capable of specifically binding to target molecules. They are selected via in vitro screening or by systematic evolution of ligands through exponential enrichment (SELEX). Aptamers fold into intricate spatial configurations, such as hairpins, convex rings, pseudoknots, and G-quadruplexes, enabling them to recognize specific antigens. Furthermore, they offer significant advantages: being animal-independent, easily synthesized with high reproducibility, and thermally stable, which allows them to be denatured and refolded repeatedly [131,133].

Aptamers have been incorporated into a wide range of sensing platforms, including electrochemical, fluorescence, colorimetric, chemiluminescent, surface plasmon resonance (SPR), and surface-enhanced Raman scattering (SER) systems. Their ease of chemical modifications enables conjugation with nanomaterials such as graphene oxide, gold nanoparticles, quantum dots, and metal–organic frameworks to improve analytical sensitivity. For aflatoxins, particularly AFB_1_, aptamer-based sensors have demonstrated excellent sensitivity and selectivity across diverse food matrices. Recent advances, such as graphene oxide-assisted SELEX (GO-SELEX) and molecular docking, have further improved aptamer affinity and target analyte recognition [130,131,133].

Comparable progress has been made in fumonisin detection, especially for FB_1_. Recent studies have demonstrated highly sensitive aptamer-based methods incorporating microfluidic systems and CRISPR-Cas technologies for fumonisin detection. For example, Zhao et al. developed a microfluidic enzyme-linked aptamer assay for rapid and ultrasensitive determination of FB_1_ in maize [134], while Qiao et al. reported a CRISPR-Cas12a-based apatasensor that that enhanced the sensitivity and selectivity of FB_1_ detection [135]. These developments demonstrate the growing potential for fumonisin monitoring alongside the established applications for aflatoxins. However, most currently available aptamer-based platforms are designed to detect free fumonisins and do not inherently address the analytical challenge posed by hidden or matrix-associated fumonisins, which often require additional extraction steps prior to analysis. The developments in aptamer technology highlight a broader trend toward portable, multiplex, and intelligent biosensing platforms capable of simultaneously detecting mycotoxins. The integration of microfluidics, nanomaterials, smartphone-assisted detection, and advanced signal amplification strategies may enable portable, rapid assessment of aflatoxins and fumonisins in complex food matrices.

#### 3.3.4. Green Analytical Chemistry

The increasing emphasis on sustainability in analytical chemistry has driven the adoption of green chemistry principles in mycotoxin analysis. These approaches seek to reduce solvent consumption, minimize hazardous waste generation, lower energy requirements, and improve laboratory safety without compromising analytical performance. The determination of aflatoxin and fumonisin has stimulated the development of miniaturized, solvent-efficient sample extraction techniques such as QuEChERS, SPME, DLLME, and other microextraction-based methodologies. Recent advances have focused on replacing conventional organic solvents with greener alternatives such as deep eutectic solvents, ionic liquids, and aqueous extraction systems. These solvents can improve extraction efficiency while reducing environmental impact [136,137]. These strategies align with global sustainability mandates, such as the European Green Deal and various U.S. initiatives, which aim to reduce laboratory waste generation and occupational hazards [137] and are particularly relevant for large-scale programs where a large number of samples may require routine analysis.

Future research is expected to further integrate green extraction procedures with advanced detection technologies such as LC-MS/MS, HRMS, immunosensors, and aptasensors. The combination of sustainable sample preparation and high-performance analytical platforms may enable environmentally friendly monitoring of aflatoxins, fumonisins, and their modified forms while maintaining the sensitivity required for regulatory compliance.

## 4. Biomarkers of Exposure and Human Biomonitoring

Obtaining accurate exposure data for aflatoxins and fumonisins remains challenging in settings where traditional epidemiological approaches are limited, primarily because these compounds are heterogeneously distributed across food supplies. Given that these toxins originate from limited dietary sources with inconsistent concentrations, traditional food diaries and questionnaires often fail to yield reliable individual-level quantitative estimates. In resource-limited settings, the logistical and cultural burden of direct food sampling further complicates data collection. Therefore, a shift toward biomarkers is essential to address these sampling errors and provide accurate, individual-level exposure data that food analysis alone cannot capture [138]. Biomarkers are specific molecular markers—either the parent compounds or their phase I and phase II metabolites—measured in body fluids or tissues to quantify exposure to toxic substances [139]. Biomarkers of exposure may be broadly classified into short-term biomarkers, which reflect recent intake over hours to days, and long-term biomarkers, which reflect exposure over weeks or months. The choice of biomarker depends on the mycotoxin’s toxicokinetics and the objectives of the biomonitoring study.

Aflatoxin biomarkers have been identified in a wide range of biological matrices, including urine, milk, blood, and various tissues, enabling targeted biomonitoring in diverse vulnerable groups. Urine is commonly used for its non-invasive collection and the presence of acute exposure markers, specifically AFM_1_, AFB_1_-N7-guanine adducts, and AFB_1_ mercapturic acid, which reflect intake over the preceding 24–48 h. The AFB_1_-lysine serum albumin adduct is used to assess chronic exposure because it can be measured in blood [140,141]. AFM_1_ in breast milk is particularly important because it serves as a biomarker of maternal dietary exposure and infant exposure during breastfeeding, making it valuable for assessing risk in one of the most vulnerable population groups [142].

The development of effective biomarkers for fumonisins is more complex than for aflatoxins due to fumonisins’ poor bioavailability and minimal metabolism [138]. This challenge is further intensified by the occurrence of modified and matrix-associated fumonisins, which may not be fully captured by conventional analytical methods. Consequently, biomonitoring provides an integrated measure of internal exposure that may better reflect the bioavailable fraction. As highly stable molecules, FB_1_—which accounts for approximately 70% of natural contamination—serves as the primary target. Although over 90% of ingested fumonisins are eliminated via feces, urine remains the most common matrix for biomonitoring, despite recovering less than 4% of the dose [143]. In addition to the direct measurement of FB_1_, fumonisin-mediated inhibition of ceramide synthase serves as a functional biomarker. The altered sphinganine-to-sphingosine (Sa:So) ratio is a sensitive indicator of sphingolipid disruption in various animal models [138]. However, its application in human studies has been less consistent due to low physiological concentrations [144]. Recent advances have explored more stable alternatives, such as the Sa1P:So1P ratio in plasma or the accumulation of FB_1_ in hair, which show promise for characterizing chronic dietary exposure [145].

Human biomonitoring studies are adopting multi-biomarker approaches that simultaneously assess exposure to multiple mycotoxins in a single biological sample [139,146]. Such approaches are particularly relevant for aflatoxins and fumonisins because they frequently co-occur in staple foods and may exert additive or synergistic toxic effects. The simultaneous determination of biomarkers provides a more comprehensive assessment of co-exposure than single analyte monitoring. At the population level, multi-biomarker strategies improve exposure characterization, facilitate identification of high-risk groups, and support the development of effective public health interventions and regulatory policies.

Human biomonitoring integrates these biomarkers into population exposure assessments; an approach that has been instrumental in identifying high-risk groups, evaluating intervention efficacy, and informing regulatory decisions. Despite these advancements, significant challenges persist. Emerging, modified, and hidden forms of aflatoxins and fumonisin often lack biomarkers or regulatory-defined limits. Additionally, inter-individual variability in absorption and metabolism, the standardization of sampling procedures, and the limited availability of robust biomarkers for chronic fumonisin exposure continue to complicate exposure assessment [140,147]. Future advances in multi-biomarker analysis are expected to improve the accuracy of population exposure assessment and strengthen risk characterization for co-exposure to aflatoxins and fumonisins.

## 5. Chemical Co-Exposure and Interaction

The toxicity of aflatoxins and fumonisins is influenced by their frequent co-occurrence in food matrices, leading to complex biochemical and cellular interactions. Experimental and epidemiological studies have reported synergistic, additive, or antagonistic effects depending on the dose, duration, and biological context of exposure [148].

### 5.1. Aflatoxin–Fumonisin Interactions

Once ingested, AFB_1_ is oxidized by cytochrome CYP450 enzymes to form several metabolites. The reactive metabolite, AFB_1_-exo-8,9-epoxide, binds to the N7 position of guanine to form DNA adducts and to albumin in serum [149,150]. FB_1_ inhibits dihydroceramide synthase (CerS), disrupting the de novo sphingolipid biosynthesis pathway and leading to the accumulation of cytotoxic sphinganine and sphingosine, which alter cell membrane integrity, cell signaling, and cell death [151,152,153].

The disruption of sphingolipid metabolism by fumonisins may potentiate aflatoxin hepatotoxicity by impairing cellular repair mechanisms and enhancing oxidative stress. This triggers the production of reactive oxygen species (ROS), which cause DNA damage. Several studies demonstrate that combined exposure to aflatoxins and fumonisins leads to increased DNA adduct formation and altered expression of apoptosis regulators, such as Bcl-2 and p53, suggesting synergistic carcinogenic potential [154,155].

Toxicokinetic differences further influence co-exposure outcomes. AFB_1_ is rapidly absorbed in the small intestine and undergoes hepatic bioactivation, whereas FB_1_ exhibits low bioavailability (<4%) but may exert prolonged effects on the intestinal epithelial cells prior to absorption. Genetic polymorphisms affecting detoxification pathways, particularly S-transferases, may further modify individual susceptibility to combined mycotoxin exposure [6,26,156,157,158].

### 5.2. Evidence from Experimental Studies

Experimental investigations have demonstrated that interactions between aflatoxins and fumonisins are highly dependent on exposure conditions and biological models. In vitro studies have reported increased oxidative stress, lipid peroxidation, DNA damage, and apoptosis following combined exposure compared with individual toxins. Similarly, animal studies have shown enhanced hepatotoxicity, altered sphingolipid metabolism, and increased incidences of preneoplastic lesions. However, not all studies report synergistic effects; additive and occasionally antagonistic responses are also observed. These findings indicate that aflatoxin–fumonisin interactions are complex and dose-dependent rather than universally synergistic [148,149,150,151,152,153,154,155].

### 5.3. Human Co-Exposure Evidence

Evidence from food surveillance studies indicates that co-exposure to aflatoxins and fumonisins is common in maize, one of the most widely consumed foods. Investigations in Brazil, Mexico, and Honduras have reported co-occurrence of both mycotoxins in maize and maize-based products, suggesting that simultaneous dietary exposure is widespread [10,159,160]. Further, intake assessments further indicate that vulnerable populations may experience chronic exposure to mycotoxins. Human biomonitoring studies complement these findings by demonstrating measurable internal exposure to both mycotoxins. The detection of aflatoxin and fumonisin biomarkers in biological samples confirms that co-exposure extends beyond contamination data and represents a genuine public health concern. Although epidemiological evidence directly linking biomarker-defined co-exposure to specific health risks remains limited, these studies demonstrate the biological likelihood of combined toxic effects observed in experimental models. These observations highlight the need for integrated risk-assessment frameworks that account for combined exposure scenarios and their potential implications for human health.

### 5.4. Implications for Risk Assessment

The significance of these mycotoxins is reflected in their classification by the International Agency for Research on Cancer (IARC). Aflatoxins are classified as Group 1 carcinogens, while fumonisin B_1_ is classified as Group 2B (possibly carcinogenic to humans) [161]. Despite evidence of frequent co-occurrence and potential interactions, regulatory frameworks generally assess these mycotoxins individually. Therefore, current approaches may underestimate risks associated with cumulative exposure. Future risk assessment strategies should therefore consider combined exposure and potential interactions to provide more realistic estimates of health risks.

**Figure 3:**
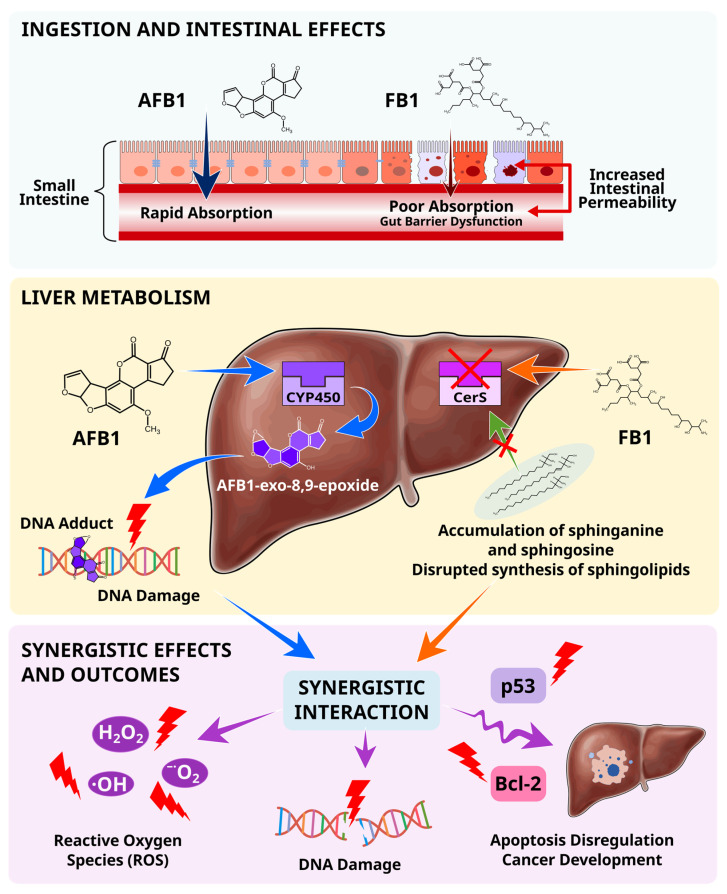
AFB_1_-FB_1_ co-toxicity pathways.

Figure 3 illustrates the complex co-toxicity pathways of aflatoxins and fumonisins, detailing the absorption differences in the small intestines, the specific liver metabolic pathways involving CYP450 and CerS and the resulting synergistic oxidative stress and DNA damage.

## 6. Impact on Human Health

Human exposure to aflatoxins and fumonisins is associated with adverse health implications ranging from acute toxicity to chronic diseases. These implications are particularly pronounced in regions where maize and other susceptible staple foods constitute a major component of the diet and food safety controls are limited. The carcinogenic significance of these mycotoxins is reflected in their classification by the International Agency for Research on Cancer (IARC), which classifies aflatoxins as Group 1 carcinogens, and fumonisin B_1_ as Group 2B (possibly carcinogenic to humans). While aflatoxins and fumonisins exert distinct toxicological effects, increasing evidence suggests that co-exposure may contribute to additive or synergistic effects [10,148,161,162].

### 6.1. Hepatocellular Carcinoma

AFB_1_ is a potent driver of liver cancer. Its biotransformation into the 8,9-epoxide leads to mutagenic aflatoxin-DNA adducts, particularly at codon 249 of the *TP53* tumor suppressor gene, resulting in characteristic G-to-T transversion mutations. This mutation compromises p53-mediated DNA repair and apoptotic pathways, thereby facilitating the development of hepatocellular carcinoma (HCC) [163,164,165]. Epidemiological evidence indicates a synergistic interaction with chronic hepatitis B virus (HBV) infection, which independently causes liver inflammation and genomic instability, thereby increasing carcinogenic risk [166,167,168]. Further evidence suggests a significant synergistic effect of AFB_1_ and FB_1_ in the pathogenesis of HCC. Historical data from high-risk regions, such as China and Guatemala, have documented frequent co-contamination with these toxins, suggesting that FB_1_ may contribute to AFB_1_-linked HCC [169]. These observations suggest that fumonisins may act as promoters in aflatoxin-associated hepatocarcinogenesis. Through the disruption of sphingolipid metabolism, induction of oxidative stress, and interference with cellular repair pathways, fumonisins may enhance the carcinogenic effects of aflatoxins, although the magnitude of this interaction in human populations remains incompletely understood.

### 6.2. Esophageal Cancer

While the liver is the primary target for aflatoxins, fumonisins have been epidemiologically linked to distinct pathologies [19]. High dietary intake of fumonisins correlates with increased rates of esophageal cancer (EC), although the underlying mechanism remains unclear. A study by Yu et al. [170] proposed the HDAC/PI3K/Akt signaling pathway as a novel mechanism for FB_1_-related esophageal cancer. Although AFG_1_ is less toxic than other aflatoxin variants, it has also been linked to esophageal cancer. A study by Li et al. [171] demonstrated that AFG_1_ can reduce the expression of HLA-I, TAP-1, and LMP-2, leading to defects in antigen presentation to T lymphocytes and thereby enhancing cancer formation. While epidemiological associations have been reported in several high-risk regions, the causal relationship between fumonisin exposure and esophageal cancer remains less well established. Further studies are required to clarify the contribution of fumonisins to esophageal carcinogenesis.

### 6.3. Neural Tube Defects

Several studies implicate fumonisin exposure as the cause of neural tube defects (NTDs). The primary mechanism of FB_1_ involves competitive inhibition of ceramide synthase due to its structural similarity to sphinganine and sphingosine, thereby disrupting de novo sphingolipid biosynthesis. This inhibition leads to the accumulation of cytotoxic sphinganine and sphingosine, 1-phosphate metabolites, and decreased sphingolipid production, thereby impairing critical cellular functions such as proliferation and apoptosis. Furthermore, FB_1_ interferes with high-affinity folate transporters, disrupting cellular folate uptake during critical windows of fetal neural development. This suggests that fumonisins may functionally mimic folate deficiency, increasing the risk of birth defects even in populations with adequate dietary folate intake. Epidemiological associations between fumonisin exposure and neural tube defects have been reported in populations with high maize consumption, supporting the evidence linking disrupted sphingolipid metabolism and impaired folate transport to abnormal fetal development [172,173].

### 6.4. Childhood Stunting, Immunity, and Gut Microbiome

The relationship between aflatoxin exposure and infant stunting involves several critical mechanisms that impair height-for-age development. Aflatoxin exposure contributes to nutritional deficiencies by damaging the intestinal lining and causing malabsorption of essential proteins, vitamins, and minerals. Aflatoxin-induced chronic inflammation disrupts metabolic processes and nutrient utilization, directly hindering essential infant growth. Aflatoxins disrupt hormones by affecting the endocrine system and growth hormone pathways. Maternal exposure to aflatoxins can transfer toxins through the placenta and breast milk, adversely affecting growth from gestation through infancy [174]. AFM_1_ is frequently detected in breast milk and serves as an important biomarker of both maternal and infant exposure. Monitoring AFM_1_ provides valuable insights into early-life exposure during development.

Exposure to aflatoxins can induce dysbiosis of the gut microbiome, reducing metabolic efficiency and, in turn, nutrient absorption. Aflatoxins possess antimicrobial properties that can attack specific microbial communities [175]. Additionally, AFB_1_ suppresses cell-mediated immunity, potentially reducing vaccine efficacy and resistance to infectious diseases, thereby intensifying the cycle of malnutrition and infection [176]. Human biomonitoring studies have reported associations between elevated aflatoxin biomarkers, particularly AFB_1_-lysine adducts, and reduced height-for-age scores in children. Although the contribution of fumonisins remains less clearly defined, fumonisin-induced disruption of intestinal barrier integrity and nutrient absorption may further intensify growth impairment in populations experiencing chronic co-exposure.

### 6.5. Acute Toxicity

Although chronic exposure poses long-term risks, acute aflatoxicosis remains a critical threat. Historical outbreaks, such as those witnessed in Kenya in 2004 and 2005, demonstrate the lethality of consuming maize with high aflatoxin levels. These events resulted in acute liver failure, jaundice, and death, with case fatality rates exceeding 39%. Evidence suggests that individuals with underlying fumonisin exposure or pre-existing liver compromise had significantly worse outcomes, reinforcing the synergistic effect in acute scenarios [177]. Although acute fumonisin is less frequently documented in humans, chronic fumonisin exposure commonly co-occurs with aflatoxin contamination in affected food systems and may contribute to overall health risks.

## 7. Conclusions

The co-occurrence of aflatoxins and fumonisins in staple foods remains an important food safety and public health concern. Accurate assessment of co-exposure is essential for effective risk management, yet it remains analytically challenging due to the contrasting physicochemical properties of these co-contaminants.

Advances in analytical chemistry have driven a transition from simple, single-analyte detection to comprehensive, multi-mycotoxin determination. Although chromatographic methods such as HPLC-FLD remain valuable for targeted applications, LC-MS/MS has emerged as the preferred method for simultaneous determination of aflatoxins and fumonisins because of its sensitivity, selectivity, and ability to analyze chemically diverse analytes. Additionally, HRMS has enabled the screening of non-targeted analytes, improving the detection of emerging and modified mycotoxins. Recent developments in green analytical chemistry and aptamer-based biosensors further demonstrate the movement toward more sustainable, rapid, and portable analytical solutions.

This review highlights that food monitoring alone may not fully capture population exposure. The occurrence of modified mycotoxins, particularly hidden and matrix-associated fumonisins, introduces additional uncertainty in exposure assessment and may lead to underestimation of dietary exposure. Therefore, integrating advanced food analysis with human biomonitoring provides a more comprehensive assessment of exposure by linking contamination data with internal biomarkers. Such approaches are essential for improving exposure assessment and supporting evidence-based food safety policies.

Despite substantial advances in analytical methodologies, important knowledge gaps remain regarding the toxicological interactions between co-occurring mycotoxins. Future research should focus on elucidating the mechanisms underlying aflatoxin–fumonisin interactions, including synergistic, additive, and antagonistic effects observed under different exposure scenarios. Equally important will be the development of harmonized analytical methods capable of capturing both free and modified mycotoxins across complex food matrices and biological samples. Advances in artificial intelligence-assisted data analysis, portable sensing technologies, and non-targeted mass spectrometry may enable earlier identification of emerging contaminants and more comprehensive characterization of real-world mycotoxin exposure. Collectively, such developments have the potential to transform mycotoxin risk assessment from a reactive process to a proactive framework that supports timely intervention, evidence-based regulatory decision-making, and improved protection of vulnerable populations.

## Figures and Tables

**Table 1:** Comparison of EU and Codex Alimentarius maximum limits for aflatoxins and fumonisins.

Mycotoxin	Food Commodity	EU Maximum Limit (µg·kg^−1^)	Codex Alimentarius Maximum Limit (µg·kg^−1^)
Aflatoxin B_1_	Peanuts for further processing	8.0	-
	Peanuts for direct human consumption/used as an ingredient	2.0	-
	Maize and maize products	2.0	-
	Maize for further processing	5.0	-
	Polished rice	2.0	-
	Rice for further processing	5.0	-
	Cereal based foods for infants and young children	0.1	-
Total aflatoxins (B_1_, B_2_, G_1_,G_2_)	Peanuts for further processing	15.0	15.0
	Peanuts for direct human consumption/used as an ingredient	4.0	10.0
	Maize and maize products	4.0	15.0
	Maize for further processing	10.0	15.0
	Polished rice	4.0	5.0
	Rice for further processing	10.0	20.0
	Cereal based foods for infants and young children	-	5.0
Fumonisins B_1_ and B_2_	Maize for further processing	4000	4000
	Maize for direct consumption	1000	2000
	Maize based breakfast cereals/snacks	800	800
	Maize based baby foods	200	200

Source: Commission Regulation (EU) 2023/915 [9] and Codex General Standard for Contaminants and Toxins in Food and Feed (CXS 193-1995) [8].

**Table 2:** Chemical transformations of aflatoxins and fumonisins.

Process	Aflatoxins	Fumonisins
Thermal treatment	Decompose at >235 °C; can undergo degradation above 150 °C with prolonged exposure (e.g., 10 min) [23].	Stable up to 180 °C; higher temperatures (≥190 °C) may cause degradation, sometimes forming derivatives such as protein-bound forms [24].
Alkaline treatment	Hydrolysis via the opening of the lactone ring to form β-keto acid that reduces fluorescence [25].	Hydrolysis via the cleavage of tricarballylic acid (TCA) to form hydrolyzed fumonisins (HFB) [26].
UV exposure	Aflatoxin B_1_ absorbs UV light at 222, 265, and 362 nm. The highest absorption at 362 nm increases the likelihood of degradation by modifying the double bond in the furan ring and fracturing the lactone ring [27].	FB_1_ and FB_2_ undergo UV degradation, although the degradation depends on irradiation conditions such as the presence of catalysts [28]
Biological treatment	Certain strains of bacteria and yeasts are used to remove and degrade aflatoxins [29]. Enzymes have also been shown to degrade them, although only a few enzyme families are known [30].	Biocatalysts convert fumonisins into less-toxic metabolites; degradation is affected by environmental conditions and the concentration of enzymes and microbes [24,31].

**Table 3:** Comparative analysis of mycotoxin extraction studies.

Method	Specific Technique/Material	Target Matrix	Analytes	Performance Metrics (LOD, Rec, RSD)	Advantages	Limitations	Reference
LLE	Standard LLE (Methanol/choroform/acidified solvents)	Various foods/feed	AFs, FBs	Performance varies with solvent ratio, acidified solvents aid FBs recovery	Simple operation	Co-extraction of interferences, time consuming, high solvent use	[33,34,35]
	DLLME (Hollow fiber)	Liquid foods	AFs, OTA	LOD: 0.04–0.06 μg·L^−1^	High preconcentration factor, uses micro-volumes of solvents	Limited to liquid samples	[34,36,37]
SLE	Standard SLE (Polar organic mixtures)	General	AFs, FBs	Performance highly dependent on solvent ratio	Simple, inexpensive, no sophisticated equipment	Labor intensive, high solvent use, non-selective	[32,38]
SPE	d-SPE (C18)	Corn	AFs, FBs, ZEN, DON	Rec: 68–120%	Rapid, reduced matrix interference	Matrix effects can persist without optimization	[39,40]
	µ-SPE (Ultrasonic assisted)	Fish feed	AFs	Rec: 80–100%, LOD: 0.42–1.2 µg·kg^−1^	Porous membrane protects sorbent from complex matrix, single step clean-up	Sorbent selectivity reduces on reuse	[41,42]
	SPME (Zinc oxide nanorods)	Food matrices	AFs	Rec: 86–99%, LOD: 0.01–0.07 µg·kg^−1^	Solvent free, good for semi-volatiles	Fragile fibers, coatings degrade, poor for non-volatile FBs	[43,44]
	MSPE (mGCB)	Corn and wheat	AFs, ZEN	Rec: Above 60%	Rapid, improved extraction and selectivity	Irreversible adsorption on carbon nanomaterials can lower recovery	[45,46]
	SBSE (MMIP-SB)	Milk, baby food	AFs M_1_, B, G	RSD: <10%	Integrate stirring and extraction, solvent efficient	Standard PDMS fail for polar analytes (require MIPs)	[47,48]
	PT-SPE (1:1 mix of graphene oxide and C18 anchored silica)	Foodstuff	AFs	Rec: 71–95%, LOD: 0.075–0.17 ng·g^−1^	Reduced solvent consumption/sample volume, shorter extraction time	Performance depends on sorbent properties	[49,50]
Energy Assisted Extraction	UAE (Ultrasound assisted)	Corn	FBs	Total time: ≈30 min	Shorter extraction time	Heat may degrade thermolabile mycotoxins, high cost	[51]
	MAE (Nano zirconia)	Food matrices	Multi-mycotoxins	Rec: 84–105%, LOD: 0.0036 µg·kg^−1^	Fast, green, low solvent consumption	Requires microwave transparent vessels, high cost	[52,53]
	PLE/ASE (Pressurized liquid/deep eutectic solvent)	Rice	AFs	Rec: 68–92%, LOD: 0.02–0.07 ng·g^−1^	Automated, in-cell filtration	High instrumentation cost, labor intensive cell preparation	[54,55]
	SFE (Supercritical CO_2_)	General	Non-polar toxins	High selectivity for non-polars	Green, gas like diffusivity	Poor for water-soluble toxins, can co-extracts matrix components	[56,57]
IAC	Standard IAC (Methanol:water 80:20)	Rice	AFs	Rec: 86–92%, LOD: 0.09–0.32 µg·kg^−1^	High sensitivity, low solvent consumption	Finite binding capacities, high cost, single use application	[58,59]
QuEChERS	Standard/Acidified (Formic acid addition)	General	AFs and FBs	Improved FB partitioning	Simultaneous extraction; fast and low solvent consumption	Often requires matrix-specific modification	[60,61,62,63]
	EMR-Lipid QuEChERS (Enhanced matrix removal)	Nuts	Multi-mycotoxins	Rec: 75–98%, LOQ: 0.05–5.0 µg·kg^−1^	Excellent lipid removal	Limited removal of non-lipid co-extractives	[64]
	MOF QuEChERS (MIL-101 (Cr))	Peanuts	AFbs	Rec: 74–98%, LOD: 0.05–0.10 µg·kg^−1^	High sensitivity, negligible matrix effect	High cost of MOF sorbent synthesis	[65]
	MWCNT QuEChERS (Fe_3_O_4_-MWCNTs@copolymer)	Grains	Multi-mycotoxins	Rec: 60–108%, LOD: 0.0011–1.3 µg·kg^−1^	High sensitivity, efficient multi-toxin analysis, low solvent consumption,	Requires specialized magnetic nanomaterials	[66]

LLE: Liquid–liquid extraction: DLLME: Dispersive liquid–liquid microextraction; SLE: Solid–liquid extraction; SPE: Solid-phase extraction; d-SPE: Dispersive solid-phase extraction; C18: Octadecylsilane; MOF: Metal organic framework; µ-SPE: Micro-solid-phase extraction; SPME: Solid-phase microextraction; MSPE: Magnetic solid-phase extraction; mGCB: Magnetic graphitized carbon black; SBSE: Stir-bar sorptive extraction; MMIP-SB: Magnetic molecularly imprinted polymer stir bar; PT-SPE: Pipette-tip solid-phase extraction; UAE: Ultrasound assisted extraction; MAE: Microwave assisted extraction; PLE: Pressurized liquid extraction; ASE: accelerated solvent extraction; SFE: Supercritical fluid extraction; IAC: Immune-affinity column; QuEChERS: Quick, Easy, Cheap, Effective, Rugged and Safe; MWCNT: Multi-walled carbon nanotubes; OTA: Ochratoxin A; ZEN: Zearalenone; DON: Deoxynivalenol; Rec: Recovery; LOD: Limit of detection; LOQ: Limit of quantification; RSD: Relative standard deviation; PDMS: Polydimethylsiloxane; MIP: Molecularly imprinted polymer. Note: Reported sensitivity values were obtained from different studies, analytes, matrices, and validation protocols. Consequently, direct comparison between analytical techniques should be interpreted with caution.

## Data Availability

No new data were created or analyzed during this study. Data sharing is not applicable.

## Abbreviations

The following abbreviations are used in this manuscript:

2D-TLC	Two-dimensional thin-layer chromatography
ACN	Acetonitrile
AFB	Aflatoxin B
ASE	Accelerated solvent extraction
Bcl-2	B-cell lymphoma 2
C18	Octadecylsilane
C8	Octylsilane
Cas	CRISPR-associated
CerS	Ceramide synthase
CO_2_	Carbon dioxide
CNBr	Cyanogen bromide
CRISPR	Clustered Regularly Interspaced Short Palindromic Repeats
CYP450	Cytochrome P450
DES	Deep eutectic solvent
DLLME	Liquid–liquid microextraction or dispersive liquid–liquid microextraction
DNA	Deoxyribonucleic acid
DON	Deoxynivalenol
d-SPE	dispersive solid-phase extraction
EC	Esophageal cancer
ELISA	Enzyme-linked immunosorbent assay
EMR-lipid	Enhanced matrix removal lipid
EU	European Union
FB	Fumonisin B
Fe_3_O_4_MWCNTs	Magnetic iron oxide multi-walled carbon nanotubes
Fe_3_O_4_@UiO-66-NH_2_	Magnetic amino functionalized UiO-66
FLD	Fluorescence detector
FWHM	Full width at half maximum
GO	Graphene oxide
GO-SELEX	Graphene oxide-systemic evolution of ligands through exponential enrichment
GST	Glutathione S-transferase
GSTM1	Glutathione S-transferase Mu 1
G-to-T	Guanine to thymine transversion
HBV	Hepatitis B virus
HCC	Hepatocellular carcinoma
HDAC/PI3K/Akt	Histone Deacetylase/Phosphoinositide 3-kinase/Protein Kinase B
HFB	Hydrolyzed fumonisins
HLA	Human Leukocyte Antigen class I
HLB	Hydrophilic–lipophilic balance
HPLC	High-performance liquid chromatography
HPLC-FLD	High-performance liquid chromatography with fluorescence detection
HPLC-UV-Vis	High-performance liquid chromatography with ultraviolet–visible detection
HPTLC	High-performance thin-layer chromatography
HRMS	High-resolution mass spectrometry
IAC	Immune-affinity column
IMPSE	Immunomagnetic solid phase
KBr	Potassium bromide
λ_max_	Wavelength of maximum absorption
LC-ESI-MS/MS	Liquid chromatography–electrospray ionization–tandem mass spectrometry
LC–MS/MS	Liquid chromatography–tandem mass spectrometry
LFA	Lateral flow assay
LLE	Liquid–liquid extraction
LMP-2	Low-molecular-weight protein 2
LOD	Limit of detection
LOQ	Limit of quantification
MA-d-SPE	Microwave-assisted dispersive solid-phase extraction
MAE	Microwave assisted extraction
MAX	Mixed-mode/anion-exchange
MCX	Mixed-mode/cation-exchange
mGCB	Magnetic graphitized carbon black
MgSO_4_	Magnesium sulfate
µ-SPE	Micro-solid-phase extraction
MIL-101 (Cr)	Materials Institute Lavoisier-101 (Chromium)
MIP	Molecularly imprinted polymer
MMIP-SB	Magnetic molecularly imprinted polymer stir bar
MOF	Metal organic framework
MRM	Multiple reaction monitoring
MSPE	Magnetic solid-phase extraction
MWCNT	Multi-walled carbon nanotubes
NaCl	Sodium chloride
NTDs	Neural tube defects
OPTLC	Over-pressured thin-layer chromatography
OTA	Ochratoxin A
p53	Tumor protein p53
PDMS	Polydimethylsiloxane
PLE	Pressurized liquid extraction
PSA	Primary Secondary Amine
PT-SPE	Pipette-tip solid-phase extraction
p QuEChERS (FATChERS)	Partitioned Quick, Easy, Cheap, Effective, Rugged and Safe
QuEChERS	Quick, Easy, Cheap, Effective, Rugged and Safe
QuEChERSERS	Quick, Easy, Cheap, Effective, Rugged and Safe-Enhanced Recovery and Sensitivity
Q-TOF	Quadrupole time-of-flight
RASFF	Rapid Alert System for Food and Feed
Rec	Recovery
ROS	Reactive oxygen species
RSD	Relative standard deviation
Sa:So	Sphinganine to sphingosine ratio
Sa1P:So1P	Sphinganine-1-phosphate to sphingosine-1-phosphate ratio
SBSE	Stir-bar sorptive extraction
SELEX	Systemic evolution of ligands through exponential enrichment
SER	Surface-enhanced Raman scattering
SFE	Supercritical fluid extraction
SLE	Solid–liquid extraction
SPE	Solid-phase extraction
SPME	Solid-phase microextraction
SPR	Surface plasmon resonance
TAP-1	Transporter associated with antigen processing 1
TCA	Tricarballylic acid
TFA	Trifluoroacetic acid
TLC	Thin-layer chromatography
UAE	Ultrasound assisted extraction
UHPLC-ESI-MS/MS	Ultra-high performance liquid chromatography–electrospray ionization–tandem mass spectrometry
UHPLC-MS/MS	Ultra-high performance liquid chromatography–tandem mass spectrometry
UPLC-Q-TOF-MS	Ultra performance liquid chromatography–quadrupole time-of-flight mass spectrometry
UV	Ultraviolet
UV-Vis	Ultraviolet–visible
WAX	Weak anion exchange
ZEN	Zearalenone
